# Corticostriatal Oscillations Predict High vs. Low Drinkers in a Rat Model of Limited Access Alcohol Consumption

**DOI:** 10.3389/fnsys.2019.00035

**Published:** 2019-08-13

**Authors:** Angela M. Henricks, Lucas L. Dwiel, Nicholas H. Deveau, Amanda A. Simon, Metztli J. Ruiz-Jaquez, Alan I. Green, Wilder T. Doucette

**Affiliations:** ^1^Department of Psychiatry, Geisel School of Medicine at Dartmouth, Hanover, NH, United States; ^2^Department of Molecular and Systems Biology, Geisel School of Medicine at Dartmouth, Hanover, NH, United States; ^3^Department of Psychological and Brain Sciences, Dartmouth College, Hanover, NH, United States; ^4^The Dartmouth Clinical and Translational Science Institute, Dartmouth College, Hanover, NH, United States

**Keywords:** alcohol, brain stimulation, local field potentials, nucleus accumbens, medial prefrontal cortex

## Abstract

Individuals differ in their vulnerability to develop alcohol dependence, which is determined by innate and environmental factors. The corticostriatal circuit is heavily involved in the development of alcohol dependence and may contain neural information regarding vulnerability to drink excessively. In the current experiment, we hypothesized that we could characterize high and low alcohol-drinking rats (HD and LD, respectively) based on corticostriatal oscillations and that these subgroups would differentially respond to corticostriatal brain stimulation. Male Sprague–Dawley rats (*n* = 13) were trained to drink 10% alcohol in a limited access paradigm. In separate sessions, local field potentials (LFPs) were recorded from the nucleus accumbens shell (NAcSh) and medial prefrontal cortex (mPFC). Based on training alcohol consumption levels, we classified rats using a median split as HD or LD. Then, using machine-learning, we built predictive models to classify rats as HD or LD by corticostriatal LFPs and compared the model performance from real data to the performance of models built on data permutations. Additionally, we explored the impact of NAcSh or mPFC stimulation on alcohol consumption in HD vs. LD. Corticostriatal LFPs were able to predict HD vs. LD group classification with greater accuracy than expected by chance (>80% accuracy). Moreover, NAcSh stimulation significantly reduced alcohol consumption in HD, but not LD (*p* < 0.05), while mPFC stimulation did not alter drinking behavior in either HD or LD (*p* > 0.05). These data collectively show that the corticostriatal circuit is differentially involved in regulating alcohol intake in HD vs. LD rats, and suggests that corticostriatal activity may have the potential to predict a vulnerability to develop alcohol dependence in a clinical population.

## Introduction

Excessive alcohol consumption is a major health concern in the United States, leading to approximately 88,000 deaths per year (Centers for Disease Control and Prevention, 2013), but only a small proportion of individuals who drink alcohol become dependent later in adulthood (Costanzo et al., 2007). A combination of environmental and genetic risk factors are associated with the development of alcohol dependence in humans, and these risk factors produce significant characteristic neurobiological effects (Hägele et al., 2014; Matošić et al., 2016). In preclinical studies, rats selectively bred to be high drinkers show neural and behavioral phenotypes related to alcohol dependence (e.g., relapse behavior, altered dopamine signaling in the striatum, etc; McBride and Li, 1998; Crabbe, 2014). Even in outbred rats, there are significant variations in alcohol intake levels, so rodent models of limited access alcohol consumption have been employed to attempt to further study the neurobiological readouts of risk factors associated with the development of alcohol dependence. Rats categorized as high or low alcohol drinkers (HD and LD, respectively) display differences in anxiety, impulsivity, and cognitive behaviors (Pratt et al., 2002; Wilhelm and Mitchell, 2008; Sharko et al., 2013), as well as differences in gene expression known to influence appetitive behavior (Morganstern et al., 2012). HD rats also show behavioral phenotypes that may be associated with an increased vulnerability to develop addiction (Spoelder et al., 2017). Additional work is needed, however, to understand how systems-level neural activity relates to the HD phenotype in rats.

Previous research in humans and rodents indicates that a history of alcohol use is associated with dysregulation in the corticostriatal circuit (Camchong et al., 2013; Broadwater et al., 2018), including the nucleus accumbens (NAc) and human medial prefrontal cortex (mPFC), referred to in rodents as the prelimbic and infralimbic cortex. The NAc integrates cortical inputs and indirectly sends feedback to the mPFC (Goto and Grace, 2008), and is particularly important in the motivating and rewarding properties of abused drugs (Koob and Volkow, 2010). The mPFC is activated in response to reward-related cues, and it has been suggested that deficits in the ability to inhibit responses to drug and associated cues arise from reduced top-down control of the mPFC to striatal regions (Goldstein and Volkow, 2002). Additionally, stimulation of the NAc has recently been used to reduce alcohol consumption in both humans and rodents (Knapp et al., 2009; Henderson et al., 2010; Voges et al., 2013), though it is important to note that brain stimulation suffers from the same highly variable treatment outcomes observed with other psychiatric treatments. However, our previous research with binge eating suggests that neural oscillations from the NAc can provide systems-level information regarding individual variability in the effect of stimulation on eating behavior (Doucette et al., 2018). The corticostriatal circuit is, therefore, an important target for studies aimed at understanding how risk factors leading to alcohol dependence become instantiated in the brain.

In the current experiment, we hypothesized that we could predict which rats were HD or LD using local field potential (LFP) oscillations recorded within the corticostriatal network, and, as a secondary aim, we assessed whether these subgroups would respond differentially to cortical or striatal stimulation. We recorded LFPs from two corticostriatal brain regions [NAc shell (NAcSh) and rat mPFC] and subsequently treated rats with high-frequency brain stimulation in each of those regions (separately) during alcohol drinking sessions. We theorized that variation in the effect of stimulation on alcohol behavior in HD vs. LD may, in part, be related to individual differences in the networks that underpin alcohol consumption.

## Materials and Methods

### Animals

Male Sprague–Dawley rats (*n* = 13) were purchased from Harlan (South Easton, MA, USA) and arrived on postnatal day 60. All animals were housed individually on a reverse 12-h light cycle with *ad libitum* access to food and water. All experiments were carried out in accordance with the National Institute of Health Guide for the Care and Use of Laboratory Animals (NIH Publications No. 80-23) and were approved by the Institutional Animal Care and Use Committee of Dartmouth College.

### Electrode Implantation

Electrodes were designed and constructed in-house and were similar to those used in our previous publication (Doucette et al., 2015). Following 1 week of habituation to the animal facility, animals were anesthetized with isoflurane gas (4% induction, 2% maintenance) and mounted in a stereotaxic frame. Custom electrodes were implanted bilaterally targeting NAcSh (from Bregma: DV −8 mm; AP +1.2 mm; ML ±1.0 mm) and mPFC (from Bregma: DV −5 mm; AP +3.7 mm; ML ±0.75 mm). Four stainless steel skull screws were placed around the electrode site and dental cement (Dentsply, York, PA, USA) was applied to secure the electrodes in place. Animals were allowed to recover for at least 1 week prior to being tethered to the recording apparatus and trained to consume a 10% alcohol solution.

### Alcohol Consumption Training

Animals were trained to drink 10% alcohol in a limited access paradigm. Three days per week (M, W, F) animals were transferred from their home cage to custom stimulation chambers, tethered at their head to stimulation cables, and given free access to 10% alcohol for 90 min. Animal weights and the volume of alcohol consumed was measured following each session in order to calculate g/kg of alcohol consumed. Animals were allowed to drink while plugged-in to stimulation cables, without stimulation, for 12 sessions.

### Local Field Potential Recordings

Prior to brain stimulation sessions (but after exposure to alcohol), animals were tethered for LFP recording in a chamber that was distinct from the alcohol consumption and stimulation chamber. Animals engaged in free behavior while tethered through a commutator to a Plexon data acquisition system and video was recorded that was time-synchronized to the LFP data (Plexon, Plano, TX, USA) during two, 30-min sessions. Noise-free data from the entire 30-min recording session were analyzed using established frequency ranges from the rodent literature (listed below) and standard LFP signal processing was used to characterize the power spectral densities (PSDs) within, and coherence between brain regions (bilateral NAcSh and mPFC) for each animal using custom code written for Matlab R2015b (described briefly below).

A fourth-order Chebychev type I notch filter centered at 60 Hz was applied to all of the data to account for 60 Hz line noise. The data was then down-sampled by a factor of five from 2 kHz to 400 Hz. A threshold of ±2 mV was used to identify noise artifacts and remove data using intervals 12.5 ms before and 1 s after the artifacts. To measure power and coherence, we used epochs of noise-free LFP data that were at least 3 s long. For epochs that were longer than 3 s, we segmented them into 3-s sections removing the remainder to keep all of the data continuous over the same amount of time.

PSDs were computed for each noise-free LFP epoch using MATLAB’s *pwelch* function using a 1.28 s Hamming window with 50% overlap. The PSDs for each 3-s segment were then averaged together to get a single representative PSD for the 30-min recording session. Total power (dB) per frequency range was calculated using the following ranges: delta (Δ) = 1–4 Hz, theta (θ) = 5–10 Hz, alpha (α) = 11–14 Hz, beta (β) = 15–30 Hz, low gamma (lγ) = 45–65 Hz, and high gamma (hγ) 70–90 Hz (McCracken and Grace, 2009; Catanese et al., 2016). To account for the 60 Hz notch filter, power values of frequencies from 59 Hz to 61 Hz were not included in the analysis. The power per frequency band was then normalized as a percent of the average total power of the signal from 1 Hz to 90 Hz (beginning of Δ to end of hγ).

Coherence was computed using the function *mscohere* with a 1.28 s sliding Hamming window with 50% overlap. The average coherence between each pair of frequency bands from 1 Hz to 90 Hz (excluding values corresponding to the 60 Hz notch filter) were used to normalize the average coherence of each frequency band within that neural site pair.

### Brain Stimulation

**Figure 1:**
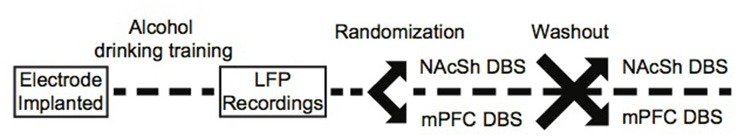
Experimental timeline.

To deliver stimulation, a current-controlled stimulator (PlexStim, Plexon, Plano, TX, USA) was used to generate a continuous train of biphasic pulses (90 μs pulse width, 130 Hz, 200 μA). These parameters produce an estimated charge density at the electrode surface below the threshold known to induce neuronal injury (Kuncel and Grill, 2004). We also chose 130 Hz stimulation because this frequency matches what was used in a clinical study of DBS for alcohol dependence (Voges et al., 2013). The output of the stimulator (current and voltage) was verified visually for each animal before and after each stimulation session using a factory-calibrated oscilloscope (TPS2002C, Tektronix, Beaverton, OR, USA). Stimulation was initiated immediately before animals had access to alcohol and turned off at the completion of the 90-min stimulation session. Animals were exposed to NAcSh or mPFC brain stimulation for three consecutive sessions in a counterbalanced fashion, followed by a washout period, such that all animals were exposed to both NAcSh and mPFC stimulation (for example, three NAcSh sessions, then washout, then three mPFC sessions). During the washout periods, animals were again allowed to drink alcohol while plugged-in to the stimulation cables but without stimulation turned on. It is important to note that a subset of animals also received stimulation to the NAcSh (*n* = 6) and mPFC (*n* = 7) at 20 Hz with washout periods between the two different stimulation parameters. We piloted the impact of low-frequency stimulation on alcohol drinking behavior because the frequency of stimulation more accurately mimics clinical transcranial magnetic stimulation (TMS), but we did not observe significant changes in drinking behavior in either stimulation target (Supplementary Figures S1A,B). Figure 1 outlines theexperimental timeline.

### Statistical Analysis

#### Categorizing Rats as HD or LD

The average alcohol consumed (in g/kg) during the last 3 days of the training drinking sessions (prior to stimulation) was calculated for each rat. Rats were subsequently categorized as HD or LD using a median split, which served as the dependent outcome used to build prediction models from the LFP features.

#### Linking Corticostriatal Activity to HD vs. LD Phenotypes

Each recording session produced 60 LFP features: 24 measures of power (6 frequency bands × 4 channels) and 36 measures of coherence (6 frequency bands × 6 channel combinations). We used a penalized regression method (lasso) in order to capture potential combinations of LFP features that correlated with behavioral phenotypes (HD vs. LD). The Matlab package *Glmnet* was used to implement the lasso using a 4-fold cross-validation with 100 repetitions. The accuracy of the model is reported as the average cross-validated accuracy. We repeated the entire above process on 100 random permutations of the data. Due to not having enough samples for a naïve test-set, we calculated the mean accuracy and 95% confidence intervals of cross-validated accuracy from the observed and permuted data distributions for comparison. If the average model accuracy outperformed chance, we implemented exhaustive single feature regressions using each LFP predictor to determine the relative information content of each feature, as we have previously described in detail (Dwiel et al., 2019).

#### Calculating the Response to Brain Stimulation

The overall effect of NAcSh or mPFC stimulation on the amount of alcohol consumed was analyzed using a RMANOVA, with average alcohol consumption (in g/kg) from the 3 days of training and the three sessions of stimulation as the within-subjects factors, and group (HD or LD) as the between-subjects factor.

### Verification of Electrode Placement

At the end of the experiment, animals were euthanized using CO_2_ gas and brains were snap frozen in 2-methylbutane on dry ice. Tissue was stored at −20°C prior to sectioning, thionine staining and histologic analysis.

## Results

### Categorization of Rats as HD or LD

**Figure 2:**
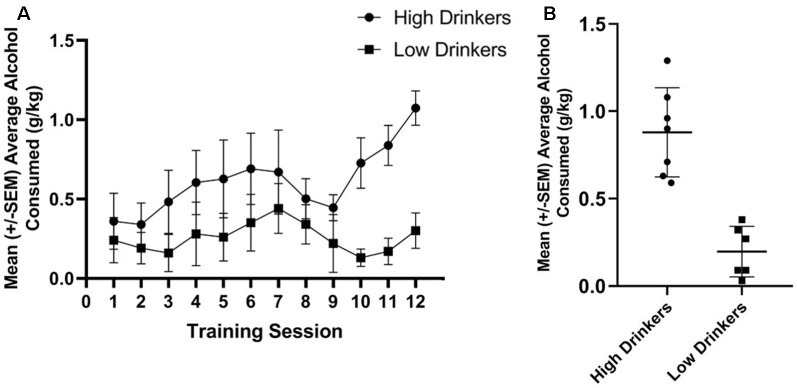
Categorization of rats as high or low alcohol drinkers (HD or LD). Average g/kg of alcohol consumed for HD and LD was significantly different across the 12 training sessions, prior to stimulation (*F*_(1,11)_ = 5.86, *p* = 0.03, $n_{\text{p}}^{2}$ = 0.35; **A**). Average g/kg of alcohol consumed across the last 3 days of alcohol drinking training was significantly different between HD and LD (*t*_(11)_ = 5.79, *p* = 0.00; **B**; *n* = 6–7/group).

Using a median split of average alcohol consumed (in g/kg) over the last three sessions of limited access to alcohol, seven rats were categorized as HD and six rats were categorized as LD. Figure 2A shows the amount of alcohol consumed during the 12 limited access sessions prior to stimulation stratified by group. Figure 2B shows average alcohol consumed during training for the HD and LD, as well as the individual variability in behavior.

### LFPs Recorded Within Corticostriatal Regions Predict HD vs. LD

**Figure 3:**
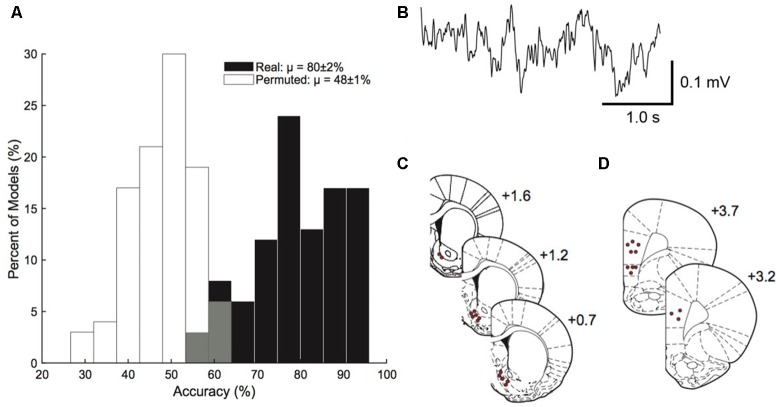
Prediction model. Corticostriatal local field potential (LFP) oscillations predict HD vs. LD better than permuted data (permuted μ = 48 ± 1%; real μ = 80 ± 2%; *n* = 10–14/group; **A**). A sample trace of corticostriatal LFP oscillations used in the prediction model **(B)**. Histology figures representing electrode placements in the nucleus accumbens shell (NAcSh; **C**) and medial prefrontal cortex (mPFC; **D**) from Bregma.

The model built from corticostriatal LFP features was able to outperform permuted data in predicting which rats were HD vs. which rats were LD (permuted μ = 48 ± 1%, real μ = 80 ± 2%; Figure 3A). A sample trace of the LFP recordings is provided in Figure 3B. Figures 3C,D show the precise placement of the electrodes. Using single feature logistic regression models, we identified the following neural features as containing significant information regarding HD vs. LD: right NAcSh lγ (increased in HD), right mPFC lγ (increased in HD), right NAcSh—left NAcSh α (coherence reduced in HD), right NAcSh—right mPFC hγ (coherence increased in HD), and left mPFC—right mPFC hγ (coherence increased in HD). Interestingly, the top 4/5 neural features important in building the prediction model indicate that γ power and coherence was increasedin HD vs. LD.

### NAcSh Stimulation Significantly Reduces Alcohol Consumption in HD

**Figure 4:**
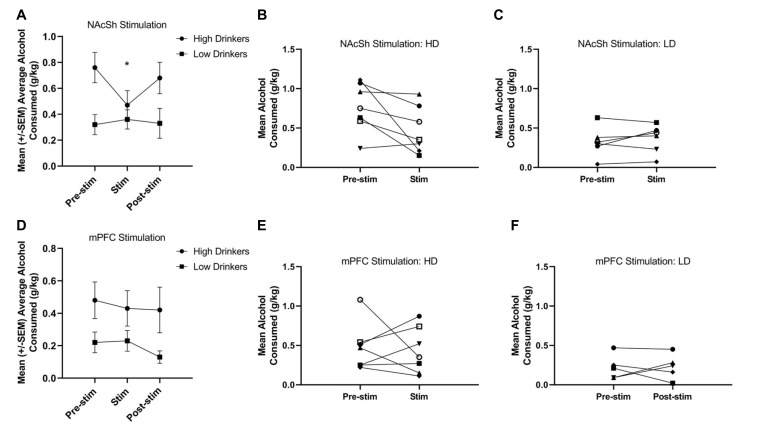
Response to 130 Hz NAcSh and mPFC stimulation. NAcSh stimulation led to a significant decrease in alcohol consumption from training (pre-stimulation) to the stimulation sessions in the HD only (*F*_(1,11)_ = 5.89, *p* = 0.03, $n_{\text{p}}^{2}$ = 0.35; **A**). Panels **(B,C)** represent the individual responses to NAcSh stimulation in HD and LD, respectively. Neither HD or LD showed a significant change in alcohol consumption due to mPFC stimulation (*F*_(1,10)_ = 0.04, *p* = 0.85, $n_{\text{p}}^{2}$ = 0.00; **D**). Panels **(E,F)** represent the individual responses to mPFC stimulation in HD and LD, respectively (*n* = 5–7/group). Asterisk denotes a significant change of *p* < 0.05.

A RMANOVA showed a significant time*group interaction (*F*_(1,11)_ = 5.89, *p* = 0.03, $n_{\text{p}}^{2}$ = 0.35) for NAcSh stimulation on alcohol consumption (Figure 4A), and suggests that HD, but not LD, showed a significant decrease in alcohol drinking due to stimulation on a population level. It is important to note, however, that individual response to NAcSh stimulation in HD was largely driven by significant changes in only a couple of rats (Figure 4B). Individual responses to NAcSh stimulation in LD suggests that this subpopulation was largely unaffected (Figure 4C).

A RMANOVA showed no effect of time (*F*_(1,10)_ = 0.04, *p* = 0.85, $n_{\text{p}}^{2}$ = 0.00) or group (*F*_(1,10)_ = 4.0, *p* = 0.08, $n_{\text{p}}^{2}$ = 0.28), nor a group*time interaction (*F*_(1,10)_ = 0.08, *p* = 0.78, $n_{\text{p}}^{2}$ = 0.01) for mPFC stimulation on alcohol consumption (Figure 4D). Figures 4E,F show the individual responses to mPFC stimulation in HD and LD, respectively. Due to electrode headcap failure, one animal in the NAc group did not receive 130 Hz mPFC stimulation.

## Discussion

Here, we show that corticostriatal oscillations can be used to classify rats as HD or LD better than chance predictions, indicating that information regarding vulnerability to excessive alcohol consumption can be extracted from neural oscillations. Additionally, when we perturbed the corticostriatal circuit with 130 Hz NAcSh stimulation, only HD rats showed a significant decrease in alcohol consumption. Interestingly, though, a closer look at the individual responses to NAcSh stimulation suggest that the significant population effect is driven by only a couple of rats. These data highlight an important caveat impeding more widespread use of circuit-based therapies (particularly invasive strategies like deep brain stimulation) in clinical populations: highly variable treatment outcomes across individuals. The fact that many of our rats did not show decreases in alcohol consumption with NAcSh stimulation suggests that perhaps not all individuals respond to stimulation of the same brain target [as we have previously observed in a model of binge eating (Doucette et al., 2018)] or the same stimulation parameters (e.g., stimulation frequency). This caveat does not necessarily diminish the impact of the current results but indicates that further advancement of circuit-based interventions requires that electrode target selection and stimulation parameters be personalized based on an individual’s unique brain structure and function. Additional research is needed to determine whether personalization of circuit-based interventions using network activity can lead to consistent and meaningful improvements in stimulation outcomes in preclinical models of addiction and other neuropsychiatric conditions.

One interesting outcome of this study is that the majority of neural features important in predicting HD vs. LD were observed within the gamma frequency range (lγ: 45–65 Hz and hγ: 70–90 Hz), where HD rats showed increased γ power and coherence compared to LD rats. Moreover, γ oscillations from the NAc have been previously correlated with reward-related behaviors in rats (van der Meer and Redish, 2009; Dejean et al., 2017), and can be altered by acute amphetamine administration (Berke, 2009). The current data suggest that γ oscillations recorded from the NAc may provide an important readout regarding vulnerability to develop excessive alcohol consumption, but future work is necessary to begin to causally relate these neural signatures with behavioral phenotypes.

Previous studies have also demonstrated that NAc stimulation can significantly reduce alcohol consumption in rodents (Knapp et al., 2009; Henderson et al., 2010). These data, in conjunction with the present experiment, suggest that NAc stimulation can reduce alcohol consumption in a subset of individuals. The NAc is an important nexus point within a complex network including reciprocal projections to frontal brain regions involved in behavioral decision-making (Goto and Grace, 2008). It is thus not surprising that the NAc is a commonly proposed target for treating addiction-related behaviors using circuit-based interventions. However, as mentioned above, future studies will need to continue to add to a growing literature linking individual variation in systems-level brain activity to variation in treatment outcomes for alcohol use disorders.

To our knowledge, the present experiment is the first to assess the efficacy of mPFC stimulation to modulate alcohol consumption in rats, though it has been investigated to alleviate treatment-resistant depression. Alcohol dependence and depression are highly co-morbid (Grant and Harford, 1995) and the neural circuits underlying both diseases significantly overlap (Pujara and Koenigs, 2014). Importantly, a recent clinical pilot study showed that TMS of the mPFC reduced craving and self-reported alcohol consumption in a group of alcoholic individuals (Ceccanti et al., 2015). While our work does not directly support the therapeutic potential of mPFC stimulation for alcohol consumption, we tested only two sets of stimulation parameters (130 and 20 Hz), and future clinical and preclinical research would need to parse this parameter space to determine the potential of mPFC stimulation targets for treating addictive disorders.

The current data set contributes to a larger literature attempting to identify vulnerable subpopulations prior to the development of addictive behaviors. At a behavioral level, HD rats show more optimal choice behavior in a gambling task, and reduced anxiety behavior in an elevated plus maze compared to LD rats (Sharko et al., 2013; Spoelder et al., 2017). These behavioral data appear to be in contrast to clinical data where alcohol use disorders are strongly associated with heightened impulsive choice and negative emotions (Boschloo et al., 2013). Both structural and functional neuroimaging techniques have also been used to identify individuals who will subsequently abuse alcohol (O’Halloran et al., 2017), and the current data supports the notion that inherent neural activity (specifically from corticostriatal circuits) prior to chronic alcohol use could be used to identify individuals at risk for becoming dependent. However, future work is necessary to investigate whether using systems-level neural activity predictors of excessive alcohol use in rodents might serve as a translational bridge to identifying vulnerable subpopulations in humans.

We would like to acknowledge several limitations in this study. First, this study is limited by a small sample size, but we used statistical methods (lasso) to shrink our predictor set and permutation controls to attempt to account for the effects of overfitting. It is important to note, however, that while our model outperformed chance, these data are correlative and there are likely other combinations of neural features that can be used to distinguish between animals that are not related to drinking behaviors. Our future work will focus on providing a causal link by attempting to change behavior by altering the specific neural features identified in this study. Second, we acknowledge that there was dorsal/ventral variation in the positioning of our electrodes with a maximum spread of ~1 mm straddling the prelimbic/infralimbic junction. Given the known confounds of volume conduction and common referencing (Bastos and Schoffelen, 2016; Carmichael et al., 2017), it is unlikely that variation in mPFC targeting meaningfully contributed to differences in the LFPs. Furthermore, the estimated diameter of the effective electric field was around ~1 mm based on previously published estimates using similar electrode designs (Hamani et al., 2014). Given that our stimulation electrode tips were at maximum 1 mm apart between PL and IL (most were closer), a significant volume of modulation from DBS would be highly overlapped. Third, though others have demonstrated that NAc or mPFC stimulation does not alter locomotor activity in rodents (Guo et al., 2013; Laver et al., 2014), we did not measure locomotor behavior during stimulation, and it may be possible that stimulation altered alcohol consumption through a non-specific mechanism (decreased activity). We also did not measure natural rewards during stimulation, but others have demonstrated that NAc or mPFC stimulation does not affect water or sucrose intake, respectively (Levy et al., 2007; Luigjes et al., 2012), suggesting that any reductions in alcohol drinking behavior we observed were not simply related to reductions in consummatory behavior in general. Finally, the current dataset only includes male rats, but males and females display significant differences in their patterns of alcohol use both in clinical populations and in rodents (Erol and Karpyak, 2015; Priddy et al., 2017). Preliminary work from our group suggests that the neural features associated with HD vs. LD phenotypes may be sexually dimorphic (unpublished findings). Therefore, our future research will focus on using the methods outlined in this experiment to determine whether sex differences in corticostriatal activity relate to sex differences in alcohol drinking behavior.

Overall, the current study is an example of how systems-level brain activity might be utilized as a tool for identifying vulnerable subpopulations to target for therapeutic interventions. Future treatments for alcohol dependence and other addictive disorders could also use similar electrophysiological and unbiased computational methods to match effective therapies to the appropriate subpopulations to decrease variability in treatment outcomes (Etkin, 2014; Doucette et al., 2018). For instance, using the methods outlined here, future work may be able to identify which individuals would show a significant response to a therapeutic intervention (e.g., DBS) using measures of brain activity, advancing our ability to improve treatment outcomes through personalization.

## Ethics Statement

All experiments were carried out in accordance with the National Institute of Health Guide for the Care and Use of Laboratory Animals (NIH Publications No. 80-23) and were approved by the Institutional Animal Care and Use Committee of Dartmouth College.

## Author Contributions

AH contributed to planning the experimental design, collected the data and wrote the manuscript. LD analyzed the LFP data, performed the computational modeling, and contributed to editing the manuscript. ND, AS, and MR-J assisted with data collection. WD and AG contributed to planning the experimental design and editing the manuscript.

## Conflict of Interest Statement

Over the past three years, AG has received research grants from Alkermes, Novartis and Janssen. He has served as an (uncompensated) consultant to Otsuka and Alkermes, and as a member of a Data Monitoring Board for Lilly. The remaining authors declare that the research was conducted in the absence of any commercial or financial relationships that could be construed as a potential conflict of interest.

## Supplementary Material

FIGURE S1 Response to 20 Hz NAcSh and mPFC stimulation. Neither 20 Hz NAcSh (*F*_(1,4)_ = 0.43, *p* = 0.85, *n*^2^_p_ = 0.01; **A**) nor mPFC (*F*_(1,4)_ = 0.79, *p* = 0.43, *n*^2^_p_ = 0.17; **B**) stimulation altered alcohol consumption from training to the stimulation sessions (*n* = 2–4/group).
