# Supplementary figures and images for: Corticostriatal Oscillations Predict High vs. Low Drinkers in a Rat Model of Limited Access Alcohol Consumption
Source: Front Syst Neurosci. 2019 Aug 13;13:35. doi: 10.3389/fnsys.2019.00035 (PMC6700217; doi:10.3389/fnsys.2019.00035)

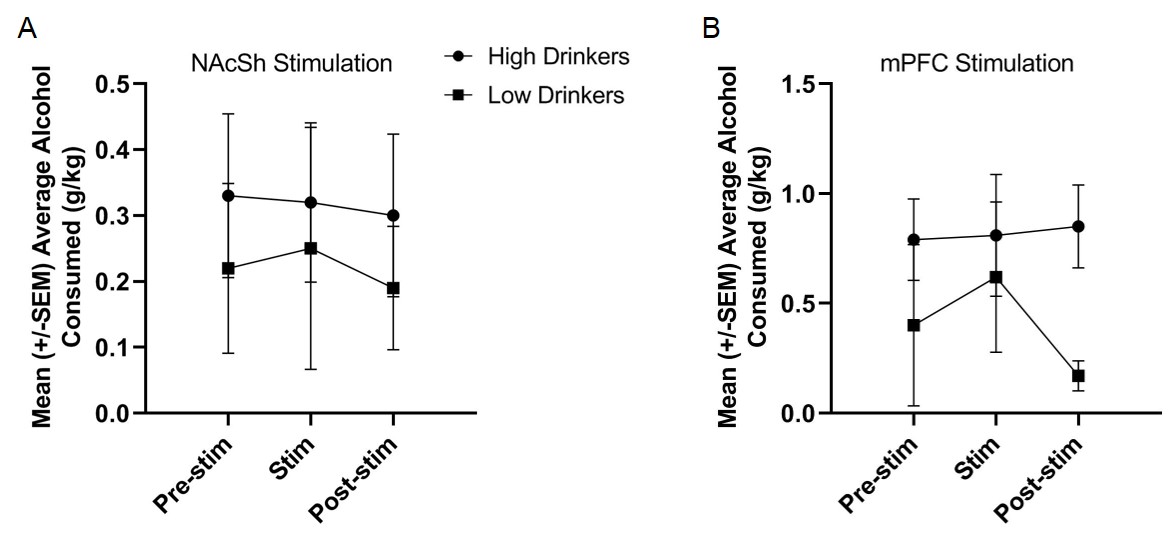

Supplement: FIGURE S1 — Response to 20 Hz NAcSh and mPFC stimulation. Neither 20 Hz NAcSh (F(1,4) = 0.43, p = 0.85, n2p = 0.01; A) nor mPFC (F(1,4) = 0.79, p = 0.43, n2p = 0.17; B) stimulation altered alcohol consumption from training to the stimulation sessions (n = 2–4/group). [file Image_1.JPEG]
